# The azygos vein pathway: an overview from anatomical variations to pathological changes

**DOI:** 10.1007/s13244-014-0351-3

**Published:** 2014-08-30

**Authors:** Sara Piciucchi, Domenico Barone, Stefano Sanna, Alessandra Dubini, Lawrence R. Goodman, Devil Oboldi, Mauro Bertocco, Cesario Ciccotosto, Giampaolo Gavelli, Angelo Carloni, Venerino Poletti

**Affiliations:** 1 Radiology Department, Morgagni Pierantoni Hospital, Forlì, Italy; 2 Radiology Department, IRCCS-IRST-Meldola, Meldola, Italy; 3 Thoracic Surgery Department, Morgagni Pierantoni Hospital, Forlì, Italy; 4 Pathology Department, Morgagni Pierantoni Hospital, Forlì, Italy; 5 Chest Radiology Department, Medical College of Wisconsin, Milwaukee, WI USA; 6 Radiology Department, San Donato Hospital, Arezzo, Italy; 7 Radiology Department, Santa Maria Hospital, Terni, Italy; 8 Pulmonology Department, Morgagni Pierantoni Hospital, Forlì, Italy

**Keywords:** Azygos vein, Inferior vena cava (IVC), Anatomical variations, Azygos continuation, Fibrosing mediastinitis, Superior vena cava syndrome

## Abstract

The azygos venous system represents an accessory venous pathway supplying an important collateral circulation between the superior and inferior vena cava. The aim of this article is to revise the wide spectrum of changes ranging from normal to pathological conditions involving the azygos system.

*Teaching points*

• *The azygos vein is a collateral venous pathway, becoming a vital shunt if major pathways of venous return are obstructed.*

• *In azygos continuation, the azygos vein becomes significantly enlarged due to inferior vena cava interruption.*

• *Fibrosing mediastinitis is an underestimated acquired disorder.*

• *Fibrosing mediastinitis induces a variable engorgement of collateral veins.*

• *Fibrosing mediastinitis leads to superior vena cava syndrome.*

## Introduction

**Fig. 1:**
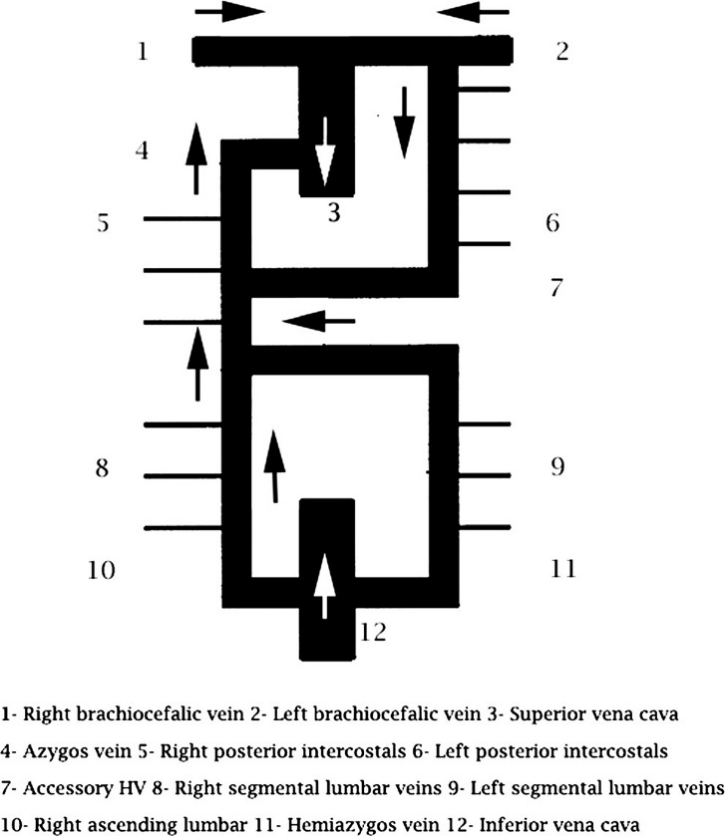
Normal anatomy of the azygos vein. The diagram shows the azygos-hemiazygos pathway

**Fig. 2:**
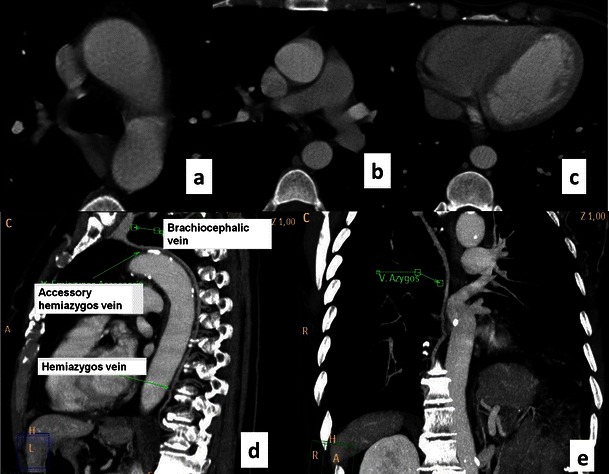
CT of the normal azygos venous system. Contrast-enhanced CT axial images show the normal appearance of the azygos vein arching from the right paraspinal space to the superior vena cava (**a**). The azygos vein runs longitudinally on the right side of the thoracic vertebral bodies (**a**). The hemiazygos vein runs on the left side of the spine (**b**) until anastomosis with the azygos vein on T8-9 as shown in the sagittal and coronal view (**c**–**d**)

The azygos, hemiazygos and accessory hemiazygos veins originate from the last portion of the posterior cardinal veins. The azygos system is a paired paravertebral venous pathway in the posterior thorax. The azygos vein originates at the junction of the right ascending lumbar and subcostal veins, entering the chest through the aortic hiatus. It ascends along the anterolateral surface of the thoracic vertebrae and arches ventrally to the right main bronchus at T5–T6, draining into the SVC. More rarely the azygos vein drains into the right brachiocephalic vein, right subclavian vein, intrapericardial SVC or right atrium [1] (Figs. 1 and 2).

The azygos system creates an important connection between the superior and inferior venae cavae. Actually, the azygos and hemiazygos systems create vital collateral pathways that become a vital shunt in cases of obstruction of the major pathways [1–3]. On the basis of Fleischner’s observation, the azygos vein, normally measuring about 0.9 cm, can be seen in 75 % of normal chest X-rays [4]. Variations in the size of the azygos vein were described by Milne and co-workers [5], who identified an azygos enlargement in the supine position or in case of overhydratation, renal failure or being in the mid-trimester of pregnancy [5–7]. Moreover, the azygos vein contributes to delineating the anatomical contours of the right paratracheal space and azygo-oesophageal recess as well. The aim of this article is to illustrate the spectrum of normal and pathological changes of the azygos venous system, with related changes of the size, flow and position.

## Congenital abnormalities

Developmental variations in the azygos system rarely cause symptoms. They are usually detected during routine examinations performed for other reasons. The most frequent congenital abnormalities include an azygos fissure, absence of the azygos vein, aortic nipple, azygos continuation of the IVC and partial venous return.

### Agenesis of the azygos vein

**Fig. 3:**
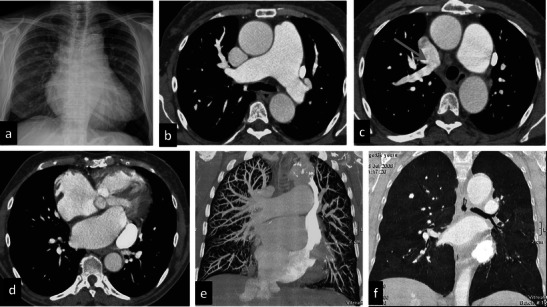
Azygos agenesis. A 64-year-old female with a prior mitral valve cardiac substitution. She underwent the PE protocol because of severe dyspnoea and hypoxaemia. Chest X-ray (**a**) shows increased pulmonary vasculature and mild dilatation of the upper mediastinum particularly on the left side and enlargement of the mediastinal pedicle. Contrast-enhanced CT shows severe enlargement of the pulmonary artery (**b**) and anomalous venous return with a right upper lobe pulmonary vein draining into the superior vena cava (**c**). On the left side a persistent left superior vena cava is seen draining into the coronary sinus (**d**), which is enlarged, too. Marked dilatation of the right atrium and ventricle can be seen. Azygos and hemiazygos veins are absent, suggesting agenesis. Coronal MIP and MinIP reconstructions (**e**–**f**) show an increase of the pulmonary vasculature (**e**) and moderate mosaic perfusion (**f**) related to severe pulmonary hypertension

The agenesis of the azygos vein (Fig. 3) occurs when the superior segment of the right supracardinal vein fails to develop. It is asymptomatic because draining of the right and left intercostal veins is guaranteed by the hemiazygos and accessory hemiazygos veins [3].

### Azygos lobe and aortic nipple

Incomplete medial migration of the right posterior cardinal vein, a precursor of the azygos vein, gives rise to an azygos lobe of the right lung in 0.4 %–1 % of the population. This anomaly can be easily indentified on CT scans, which show the azygos vein more laterally than the usual anterior arching before entering the superior vena cava (SVC) or right brachiocephalic vein. An aortic nipple can be identified when the left superior intercostal vein, draining the left second, third and fourth posterior intercostal veins, connects to the left brachiocephalic vein, forming the aortic nipple. It can be seen on a frontal radiograph as a small soft-tissue density adjacent to the lateral border of the aortic knob in about 10 % of normal patients. Contrast-enhanced chest CT images show a curvilinear contrast-filled vessel along the left lateral border of the aorta that can usually be traced from the left brachiocephalic vein to the region of the accessory hemiazygos vein [3].

### Azygos continuation

**Fig. 4:**
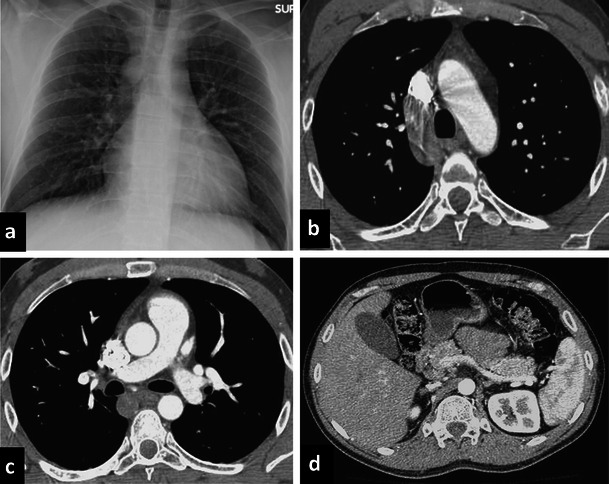
Azygos continuation. Chest X-ray shows enlargement of the azygos vein (**a**) on the right superior mediastinum. Axial enhanced CT shows an enlargement of the azygos vein (**b**–**c**) and absence of the inferior vena cava with azygos continuation (**d**)

Failure of the union between the hepatic and prerenal segments during embryological development results in the so-called “infrahepatic interruption of the inferior vena cava (IVC) with azygos continuation” (Fig. 4). Consequently, blood is shunted from the supra-subcardinal anastomosis through the retrocrural azygos vein, which is usually mildly dilated. The prevalence of azygos continuation is about 0.6 % [4].

Even though it is being increasingly identified in otherwise asymptomatic patients, azygos continuation can be observed in association with severe congenital heart disease, asplenia or polysplenia syndromes [5].

Diagnosis of this vascular anomaly is needed before cardiac catheterisation, especially in case of interventional procedures such as balloon dilatation, stent implantation or umbrella placement. It is also important before certain surgical procedures such as ligation of the azygos vein during thoracotomy or portocaval decompression surgery [8, 9]. On chest X-ray, azygos continuation can be suspected in the presence of a focal enlargement of the right paratracheal stripe above the right mainstem bronchus. A CT scan confirms the mild enlargement of the azygos vein and azygos arch secondary to increased flow. Moreover, in azygos vein continuation the infrahepatic portion of the IVC is absent [5].

## Acquired abnormalities

Acquired anomalies of the azygos vein include enlargement secondary to haemodynamic changes (fluid overload, increase in right atrial pressure, fibrosing mediastinitis and caval syndrome) or the presence of lesions (cancer, adenopathy or benign lesions), which cause compression or intraluminal filling defects. Moreover, devices in the vena cava or dislocated in the azygos vein can potentially induce thrombosis or fistulas involving the azygos lumen.

### Fluid overload and an increase in mean right atrial pressure

**Fig. 5:**
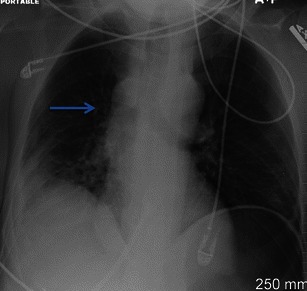
Fluid overload. A 39-year-old female with a history of transfusion after severe abdominal bleeding. AP portable chest X-ray shows an enlargement of the azygos vein (blue arrow) related to fluid overload. In the right lower basal region, a parenchymal consolidation can be seen

In physiological conditions, the microvascular hydrostatic pressure measures approximatively 7 mmHg, whereas the perimicrovascular pressure of elastic return measures about -8 mmHg. The combination of these two pressures generates hydrostatic pressure of filtration of about 15 mmHg, which is partially blanced by a colloid-osmotic pressure of 14 mmHg. A final filtration pressure of about 1 mmHg favours the continuous transvascular flow of water and solutes. The lymphatic pulmonary draining system allows this draining of water and solutes, removing the excess fluid and avoiding fluid overload in the lung. Increased hydrostatic pressure, reduced colloid-osmotic plasmatic pressure or a capillary membrane lesion may induce pulmonary oedema as a result of a change in the physiological balance of fluid. Increased hydrostatic capillary pressure is the main reason for pulmonary oedema and can be the result of left cardiac diseases or excessive fluid infusion of iso-oncotic fluids. On chest X-ray, variation in the size and contour of the azygos vein and mediastinal pedicle provides useful haemodynamic information [5–7]. The azygos vein and vascular pedicle correlate significantly with the intravascular fluid circulating pool. In fact, increased capillary blood volume (CBV) following over-infusion or renal failure, with consequent fluid overload (Fig. 5), results in a concomitant increase in the width of the azygos and vascular pedicle. Each variation of 0.5 cm on the vascular pedicle corresponds to an increase of 1 l of circulating fluid. The width of the azygos vein does not correlate significantly with the CBV, but with the right mean atrial pressure (r = 0.74; p < 0.001). These reversible relationships have been observed in patients with any degree of severity of cardiac failure and CBV values ranging from normal to very high [8–10]. Actually, Milne and co-workers [8] reported that the main reason for enlargement of the azygos vein is the increased mean right atrium pressure. Usual causes of increased mean right atrial pressure include constrictive pericarditis, cardiac tamponade, pulmonary hypertension and portal hypertension.

### Fibrosing mediastinitis

**Fig. 6:**
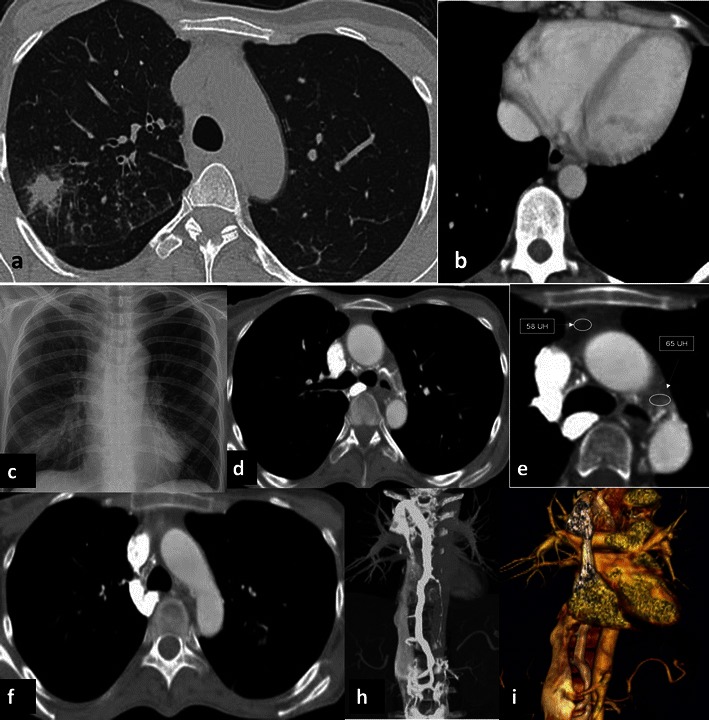
Atypical mycobacterial infection: induced fibrosis mediastinitis. A 22-year-old female, affected by celiac disease, underwent CT for fever and cough. It showed a consolidation and tree-in-bud pattern in the right upper lobe (**a**). The heart and azygos vein were normal (arrow) in size (**b**) . Bronchoscopy identified an atypical mycobacterial infection. One year later, she underwent a chest X-ray, which showed an enlargement of the superior mediastinum even though the heart size was normal (**c**). Contrast-enhanced CT scan showed a significant enlargement of the azygos vein, not present in the prior exam (**d**–**f**), associated with an increased density of the mediastinal tissue (**e**). A retrograde, early opacification of the azygous vein can be seen in the axial (**d**–**f**) and coronal MPR (**h**) and VR (**f**). In the coronal view, the inferior vena cava was enlarged as well. The patient had a fibrosing mediastinitis caused by an atypical mycobacterial infection that induced a reduced calibre of the superior vena cava in the infra-azygos region and retrograde flow in the azygos system

**Fig. 7:**
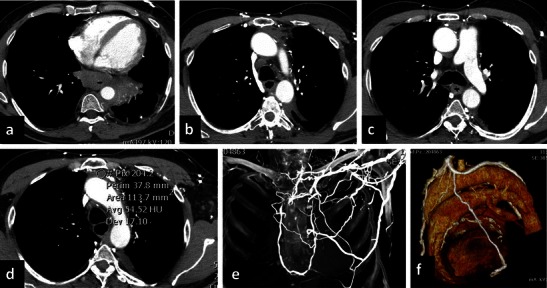
Radiation-induced fibrosing mediastinitis. A 58-year-old male treated 5 years previously with radiotherapy for mediastinal bulky lymphoma. The patient reported dysphagia, and a PET scan showed an uptake in the distal portion of the oesophagus. The CT scan shows a pathological thickening in the oesophagus and a parenchymal consolidation in the left lower lobe (**a**). Moreover, CT shows retrograde opacification of the azygos vein, hemiazygos vein (**b**, **c**) and aortic nipple. Increased density (**d**) is seen in the mediastinal tissue, particularly in the anterior mediastinum. Multiple collateral pathways were opacified, as confirmed by coronal MIP (**e**) and VR (**f**). These findings are consistent with fibrosing mediastinitis secondary to radiotherapy

**Fig. 8:**
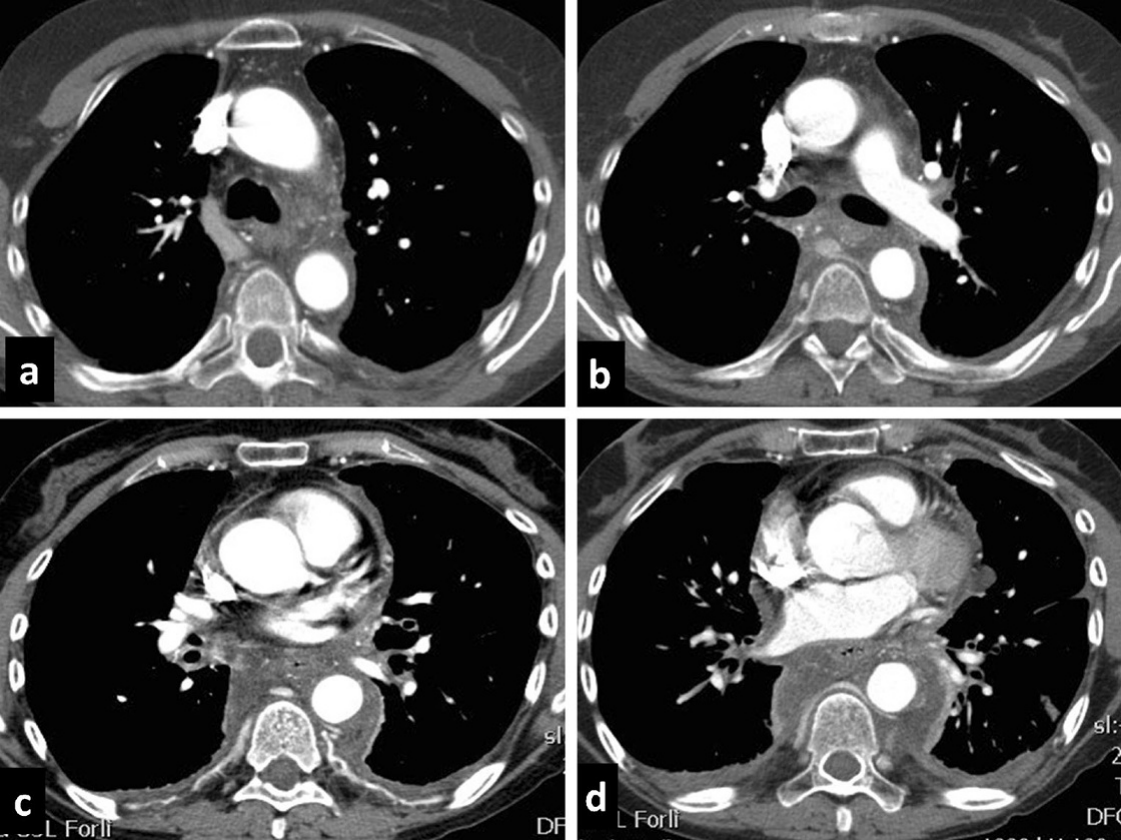
IgG4-related fibrosing mediastinitis. A 60-year-old female with a history of recurrent bilateral pleural effusion in the last 5 years. Clinical history of non-differentiated collagen vascular disease treated with steroids. CT showed marked hypertrophic tissue of the mediastinum and of the pleural space characterised as IgG4 sclerosing disease. A significant hypertrophy of the bronchial arteries (**a**–**c**) and intercostal vessels (**c**) can be observed. The azygos vein diameter is normal before anastomosis with the vena cava, whereas it shows a compression of the lumen in the medio-thoracic tract. Hypodense tissue is seen in the pleural space at the costophrenic angles

Fibrosing mediastinitis is a rare, relatively benign disorder caused by the proliferation of acellular collagen and fibrous tissue within the mediastinum [11, 12]. Patients are typically young, although the disease is reported to occur over a very wide age range. Fibrosing mediastinitis has been described as a form secondary to infections such as histoplasmosis and atypical mycobacterial infection (Fig. 6), an autoimmune disease such as Behçet’s disease or rheumatic fever; it can follow radiation therapy (Fig. 7), be related to Hodgkin’s lymphoma, be induced by drug therapy or be a manifestation of IgG4-related lymphoplasmocytic and fibrosing disorder (Fig. 8). The process may occur as a localised mass or may diffusely infiltrate the mediastinum. In rare cases it extends to the soft tissues of posterior mediastinum and pleural space, bilaterally (Fig. 8). The right side of the mediastinum is more commonly involved than the left. Although chest X-rays of patients with fibrosing mediastinitis usually appear abnormal, the findings can be quite subtle and the extent of mediastinal involvement is frequently underestimated. Chest X-ray may show signs of pulmonary venous obstruction manifesting as a localised pulmonary venous hypertension with peribronchial cuffing, septal thickening and localised oedema. CT scans show an infiltrative mass of soft-tissue attenuation that obliterates normal mediastinal fat and encases adjacent structures. Contrast-enhanced CT depicts the level and entity of the stenosis and also clearly shows collateral vessels within the mediastinum and chest wall. Calcification within the fibrous tissue is not uncommon, particularly in patients with histoplasmosis. In fibrosing mediastinitis, the azygos pathway shows variable engorgement of all the collateral veins due to the soft tissue infiltration of the mediastinum and to the related superior vena cava syndrome [12].

### Superior vena cava syndrome

**Fig. 9:**
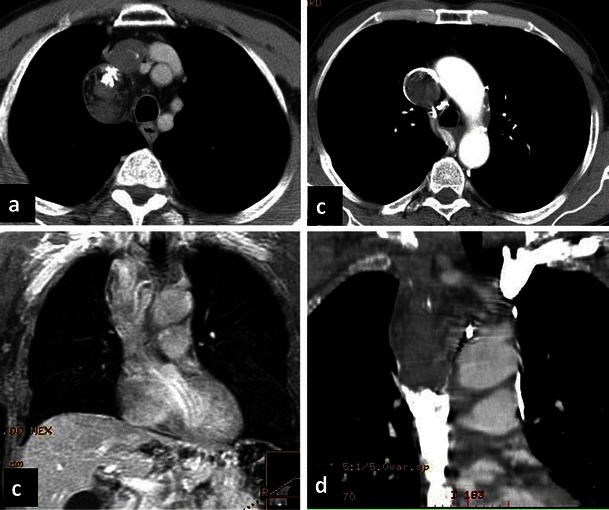
Teratoma. Non-contrast and contrast-enhanced CT (**a**, **b**) shows a rounded nodule, with some intralesional fatty spots and calcifications in the right paratracheal space consistent with teratoma. The benign lesion induced extrinsic compression of the superior vena cava

**Fig. 10:**
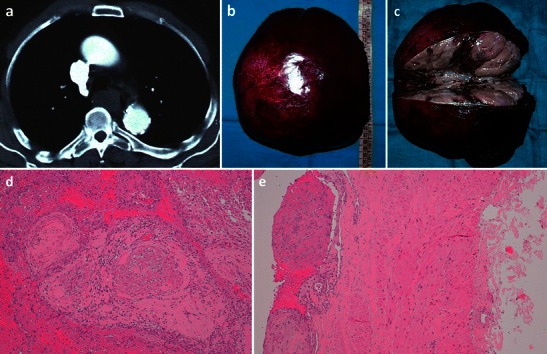
Masson’s tumour of the azygos vein. An 80-year-old man who presented with significant dysphagia and hemoptysis, in the absence of any oesophageal diseases. CT revealed a hypodense lesion measuring about 3.5 cm (**a**) in the azygo-oesophageal recess. With videothoracoscopy the solid lesion (**b**) of the distal third of the azygos vein, close to the its distal cervical branches, was resected. Macroscopic examination (**c**) showed an aneurysmatic dilatation of the azygos segment with a luminal thrombus adherent to the vein wall. Histological analysis shows multiple foci of intravascular papillary endothelial hyperplasia were present (**d**–**e**)

SVC syndrome occurs in the presence of an obstruction of blood flow through the SVC to the right atrium resulting in a severe decrease in venous return from the head, neck and upper extremities. Obstruction of the SVC can be caused by an intraluminal obstruction (thrombus, stenosis or indwelling foreign body) or external compression (tumour) (Figs. 9 and 10). SVC syndrome develops when the ability of collateral blood vessels to compensate for the SVC obstruction is exceeded [13–15]. The azygos system is the most important pathway for decompression of SVC obstruction. CT diagnosis includes two important findings: a lack or decreased opacification of central venous structures distal to the site of obstruction and intense opacification of collateral venous vessels. As obstruction is chronic, several venous collateral pathways usually develop, allowing blood to return to the right atrium. The various patterns of venous collateral pathways differ depending on the level of obstruction. The three major collateral pathways are the vertebral-azygos-hemiazygos pathway, internal and external mammary pathways, including the superficial thoracoabdominal veins, and left renal-hemiazygos pathway, including the gonadal and ureteral veins. In superior vena cava syndrome, the azygos vein may be filled by anterograde or retrograde flow. If the obstruction is above the azygos vein, the flow direction will be retrograde. If the lesion is below the azygos vein, the flow direction will be anterograde [14, 15].

### Inferior vena cava obstruction

Several causes can induce inferior vena cava obstruction, but the most important are membranous obstruction, Budd-Chiari syndrome (BCS) and neoplastic lesions [16]. In the presence of IVC obstruction, intrahepatic venous, portocaval and spider web collateral vessels develop to decompress the liver parenchyma. Extrahepatic collateral vessels commonly seen in membranous obstruction of intrahepatic IVC include the ascending lumbar veins, which drain into the azygos vein on the right side and into the hemiazygos vein on the left. Nonvascular lesions may be found next to the azygos vein, particularly in the right paratracheal region or in the azygo-oesophageal recess (Fig. 9), mimicking an enlargement of the azygos vein. Lung cancer adenopathy, sarcoidosis and lymphoma are the most common causes of enlargement of the right inferior paratracheal region. Other less common lesions are cysts of the pericardium and primary benign or malignant tumours [17].

### Insertion of devices into the azygos vein

**Fig. 11:**
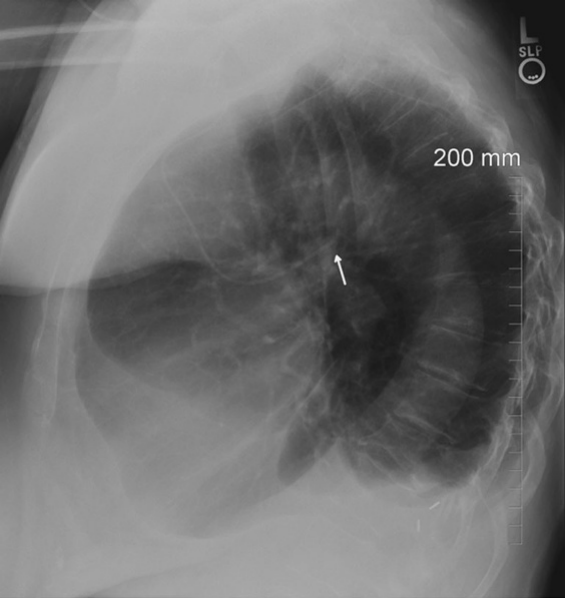
Catheter tip in the azygos vein. Lateral view of the chest X-ray shows the tip of the catheter oriented posteriorly, suggesting dislocation in the azygos vein

**Fig. 12:**
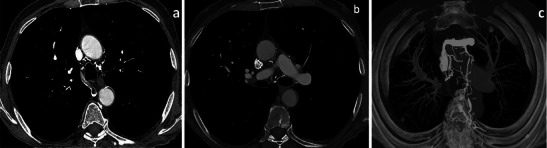
Thrombosis of the azygos vein. Thrombosis of the azygos vein (**a**) in a patient with thrombosis around the catheter tip with extension to the azygos vein (**b**). A marked hypertrophy of collateral vessels due to the shunt is seen (**c**)

Prolonged use of central devices, including central venous catheters and peripheral inserted central catheters (PICCs), can be complicated by early complications, such as pneumothorax and haemorrhage, and long-term complications, such as the development of thrombosis, infection or mechanical failure [16–18]. The tip of central devices should be placed in the SVC between the azygos vein arch ending and atriocaval junction. Its correct placement after implantation is always confirmed by repeated X-rays. The tip of the catheter can sometimes be seen inside the azygos vein; on chest X-rays the catheter can be identified as a loop in the right superior mediastinum. The lateral view of the thorax is useful in the identification of dislocation in the azygos vein because the tip is oriented posteriorly (Fig. 11). Most common complications of implantable devices are thrombosis around the tip (Fig. 12) and fistulas. Fistulas between the azygos vein and airways due to friction effects between the vein and airways have been described in the literature [19, 20]. Pacemaker implantation in the azygos vein can also induce superior vena cava syndrome [21] because of thrombosis of the tip; this is often completely asymptomatic in patients. Goudevenos and co-workers [21] found 1 out of 3,100 patients suffered from SVC syndrome after transvenous pacemaker implantation.

## Conclusions

Azygos vein changes may be associated with “quantitative” aspects such as variations in the mean right atrial pressure or qualitative changes such as the (mostly not clinically significant) anatomical congenital variants. However, conventional radiology may be the first crucial step leading to suspicion of azygos pathway changes (especially in the case of azygos continuation, haemodynamic changes or catheter misplacement). Only by knowing the diagnostic keys to these relatively rare variations can we suspect the variation and successively confirm the diagnosis with contrast-enhanced CT scans.
